# A retrospective study on the effects of exclusive donor human milk feeding in a short period after birth on morbidity and growth of preterm infants during hospitalization

**DOI:** 10.1097/MD.0000000000007970

**Published:** 2017-09-01

**Authors:** Eun Jeong Kim, Na Mi Lee, Sung-Hoon Chung

**Affiliations:** a Department of Pediatrics, Kyung Hee University School of Medicine; b Department of Pediatrics, Chung-Ang University Hospital, College of Medicine, Chung-Ang University, Seoul, Korea.

**Keywords:** donor human milk, growth assessment, infant, morbidity, very low birth weight

## Abstract

The risks and benefits of feeding preterm formula (PF) versus donor human milk (DHM) in preterm infants are uncertain, and studies evaluating the efficacy of DHM to the morbidities and growth of preterm infants in the neonatal intensive care unit are confused by the need for milk fortification. We aimed to determine and compare the outcome of short-term morbidities in neonatal intensive care unit and growth between premature infants fed exclusively DHM only until a volume of 130 mL/kg/d of enteral feeding was achieved and infants fed with a PF mix after birth. The data of 132 infants with low birth weight of <1500 g and gestational age of less than 32 weeks were considered. Ninety infants were analyzed, of which 86 were discharged alive. The DHM group (n = 36) was made up of infants who were fed exclusively with DHM, whereas the PF group (n = 54) consisted of infants who were fed with a combination of PF and either DHM or human milk, until a volume of 130 mL/kg/d of enteral feeding was achieved. Once feeding in the DHM group progressed to volumes greater than 130 mL/kg/d, infants were fed fortified DHM and PF alternately. One infant (2.8%) in the DHM group had late-onset sepsis or necrotizing enterocolitis compared with 21 (38.9%) in the PF group (adjusted odds ratio 0.05, 95% confidence interval 0.01–0.41); 13 (36.1%) infants in the DHM group had bronchopulmonary dysplasia compared with 38 (70.4%) in the PF group (odds ratio 0.18, 95% confidence interval 0.05–0.41). Although the DHM group demonstrated a comparatively lower rate of weight gain, head circumference increment, and height increment from birth to the age at which an enteral feeding volume of 130 mL/kg/d was achieved, there were no significant differences in these values at 36 weeks’ postmenstrual age between both groups.

## 1 Introduction

Breast milk provides the ideal nutrition and protective immune components that infants need for healthy development. The benefits of feeding premature infants with their own mother's raw milk are well-documented. These include decreased incidences of necrotizing enterocolitis (NEC), late-onset sepsis (LOS), feeding intolerance in preterm infants,^[1–7]^ and also lower rates of hypertension and cardiovascular risk in adolescence and adulthood.^[8]^ For maximum clinical benefits, when a mother's own milk is unavailable or insufficient for numerous reasons, the use of donor human milk (DHM) should be the first alternative, particularly for premature infants, instead of artificial formula milk.^[9–11]^

However, because the processing of a donor's milk for a milk bank requires pasteurization, freezing, and thawing for final use, the biological components of the milk, responsible for the beneficial effects, may be inactivated, resulting in the slow growth of infants. In a bid to combat the loss of the important biological components of the donor's milk, many studies have recommended the need for milk fortification.^[12–14]^ However, we are unsure of the optimal period for provision of a high-calorie nutritional support.

The objective of this study was to assess and compare the impact of 2 different groups of milk feeds (DHM and preterm formula [PF]) until a volume of 130 mL/kg/d of enteral feeding, on the growth and morbidity of hospitalized preterm infants.

## 2 Methods

### 2.1 Subjects

In this retrospective study, we reviewed 132 infants of birth weights (BWs) <1500 g and gestational ages (GAs) of less than 32 weeks, who were born alive and admitted to the neonatal intensive care unit (NICU) of the Kyung-Hee University Hospital at Gangdong in Korea, between January 2011 and December 2016. Data were gathered from the electronic medical record charts of the infants, and this included prenatal complications, delivery information, demographics, maternal obstetric and neonatal outcomes, and also data from the NICU, until either the discharge or death of the infant. The IRB waived the requirement to obtain the informed consent requirements for this retrospective chart review (approved number KHNMC 2017-03-007).

**Figure 1:**
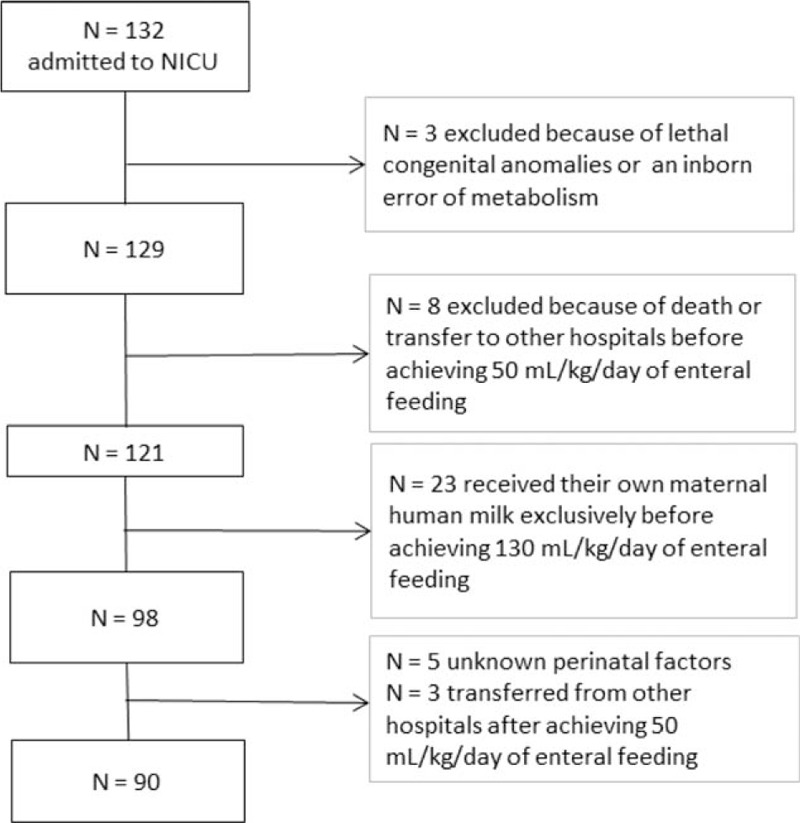
Flowchart identifying the study population.

Of the 132 infants reviewed, 3 had lethal congenital anomalies or an inborn error of metabolism and 8 infants had either died or were transferred to other hospitals before achieving a daily volume intake of 50 mL/kg/d through enteral feeding. Twenty-three infants had been exclusively breastfed with their own maternal human milk (HM) before achieving 130 mL/kg/d of enteral feeding, 5 infants were identified with unknown perinatal factors, and 3 infants were transferred from other hospitals after receiving 50 mL/kg/d of enteral feeding. All these infants (42 in all) were excluded from the study (Fig. 1). Congenital anomalies were coded according to the International Classification of Diseases (10th revision, Clinical Modification) and classified as inevitably lethal, acutely life-threatening, or not acutely life-threatening.

The study groups were divided into 2, based on whether the infants were fed exclusively with DHM (group DHM), or with a combination of a PF and either DHM or HM (group PF), before achieving an enteral intake volume of 130 mL/kg/d. Group DHM received pasteurized DHM from the HM bank (HMB) of the Kyung-Hee University Hospital at Gangdong until a volume of 130 mL/kg/d of enteral intake was achieved, then progressed to an alternating intake of DHM fortified with HM fortifier and an increased calorie PF before discharge. Group PF received mainly PF from day 1 of delivery, until discharge. PF was used as an alternative, if their maternal HM was not available. In the PF group, the percentage of DHM or HM did not exceed 10% of total enteral intake until a volume of 130 mL/kg/d of enteral intake, and 13 (24.1%) premature infants were fed exclusively with PF.

### 2.2 Maternal and neonatal variables

Perinatal variables assessed were the maternal age, preterm premature rupture of membranes (pPROM), antenatal steroids, caesarean section, multiple gestation, sex of the infants, GA, BW, small for GA, and Apgar score at 5 minutes.

Clinical variables were the use of surfactant, pneumothorax, pharmacological or surgical treatment of patent ductus arteriosus, bronchopulmonary dysplasia (BPD), retinopathy of prematurity (ROP) (≥stage 2), intraventricular hemorrhage (IVH; grade III or IV in the classification by Papile et al), LOS, and NEC (≥stage 2). Other hospital management factors assessed were the duration of a ventilator care and a central nervous catheter, postnatal ages at which enteral feeding volumes of 50 and 130 mL/kg were achieved, average length of hospital stay, and postmenstrual age (PMA) at discharge and mortality.

Bronchopulmonary dysplasia was defined as the need for supplemental oxygen dependence at 36 weeks’ PMA.^[15]^ Moderate BPD was defined, based on the diagnostic criteria of the National Institute of Child Health and Human Development Workshop severity, which requires provision of oxygen for more than 28 days, and an addition of <30% oxygen at 36 weeks’ PMA. Severe BPD was defined as the need for oxygen for >28 days plus ≥30% oxygen and/or positive pressure at 36 weeks’ PMA, according to National Institute of Child Health and Human Development criteria.^[16]^ NEC was defined based on a modified Bell staging classification grade ≥II. LOS was defined as a positive blood culture from a peripheral artery or vein after 5 days of life, in symptomatic infants with antibiotic treatment.^[17]^

During hospitalization, the body weight was measured in grams at a specific time each day, using digital scales. The head circumference (HC) and the crown-heel length were measured every Monday, and at 36 weeks’ PMA.

### 2.3 Nutrition management

The same protocol for parenteral nutrition and feeding was followed for both groups. The differences existed in the type of milk fed to each group. Amino acid (AA) was administered parenterally, at a rate of 3.0 g/kg/d for all infants on the first day of life. From the second day, the AA dose was increased by 0.5 g/kg/d, up to the target amount of 4.0 to 4.5 g/kg/d, in the form of an individualized parenteral nutrition solution. Dextrose was administered with the aim of maintaining blood glucose concentration between 80 and 150 mg/dL. Twenty per cent intravenous lipid solution (Lipision, Choongwae Pharma Corp, Seoul, Korea) was administered on the second day of life at a rate of 0.5 g/kg/d, and was increased by 0.5 g/kg/d until a target amount of 3.0 g/kg/d was reached.

A standard feeding protocol introduced to our NICU was followed during the administration of feed for both groups and was not modified in any way in the course of the study (Table 1). Trophic feeding or minimal enteral nutrition (BW <1000 g, 0.5 mL every 6 hours; 1000–1499 g, 10–15 mL/kg/d) was initiated within 24 hours of life, if the infant could tolerate enteral feeding. Modification of diet commenced as soon as the infants could tolerate volumes greater than 130 mL/kg/d and were at least after 2 weeks old; infants in group DHM progressed to an alternation between DHM fortified with an HM fortifier (Maeil HMF; Maeil Dairies Co., Ltd.) and PF (Maeil Babywell Preemie, 24 kcal/oz), to enhance adequate intake of nutrient, such as protein, calcium, and phosphorous, before discharge. However, group PF continued to receive mainly PF, alternated with fortified HM if tolerated, before discharge. For all infants, after complete enteral tube feeding was achieved, the feeding volume was gradually increased until a maximum of between 150 and 160 mL/kg/d was reached, to ensure a weekly weight gain of at least 100 g/kg. The amount of intravenous fluid was concomitantly reduced as enteral feeding progressed, and parenteral nutrition supplementation stopped, when feeding enteral feeding volume reached 130 mL/kg/d.

**Table 1:**
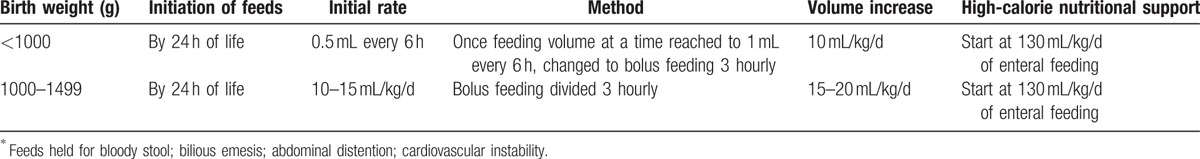
Feeding protocol at Kyung-Hee university hospital in Gangdong^∗^.

### 2.4 Human milk bank

Donor human milk was obtained from an established HMB in the Kyung-Hee University Hospital at Gangdong in Korea, which follows strict guidelines for screening, storage, and handling procedures to optimize its composition and ensure its safety for the recipient.^[18]^ The HMB was opened in August 2007, and is a nonprofit milk bank, primarily for hospitalized infants. The bank accepts donations of surplus milk from healthy lactating mothers. The HMB provides pasteurized DHM by prescription or upon a physician's order to hospitals and outpatients throughout Korea.

### 2.5 Statistical analysis

All analyses were performed using the software package SPSS version 20.0 (IBM Corp., Armonk, NY). Continuous variables of both patient groups were compared, using the Student *t* test or Mann–Whitney *U* test, and the results were expressed as means ± standard deviations (SDs). Categorical variables were compared using the chi-square test or Fisher exact test, and results were expressed as numbers and percentages. Univariate and multivariate logistic regression models were used to estimate odds ratios (ORs) and 95% confidence intervals (CIs). Multivariate logistic regression analysis was used to assess the relationship between different outcome measures and the effect of DHM feeding to the PF feeding, after adjustment for the sex, GA at birth, pPROM, and 5-minute Apgar score. Univariate analyses were used to evaluate the influence of each type of milk on growth, and expressed as z-score changes from birth at any instance at which growth data were assessed. A *P* value of <.05 was considered statistically significant.

## 3 Results

After application of the inclusion and exclusion criteria, 90 infants were analyzed, of which 86 were discharged alive, 1 was transferred to another hospital, and 3 died in the NICU. The DHM group consisted of 36 infants, whereas the PM group consisted of 54 infants.

### 3.1 Clinical characteristics of DHM and PF groups at NICU discharge

A comparison of perinatal characteristics in the DHM and PF groups is shown in Table 2. There was no significant difference in the perienatal characteristics (maternal age, maternal hypertension, use of antenatal steroids, mode of delivery, multiple gestations, sex, small for GA, 5-minute Apgar score, mean GA at birth, and mean BW) between both groups that could have influence morbidity/mortality, or clinical course of preterm infants. Compared with the PF group, mothers in the DHM group were more likely to have pPROM (50.0% vs 22.2%; *P* = .006).

**Table 2:**
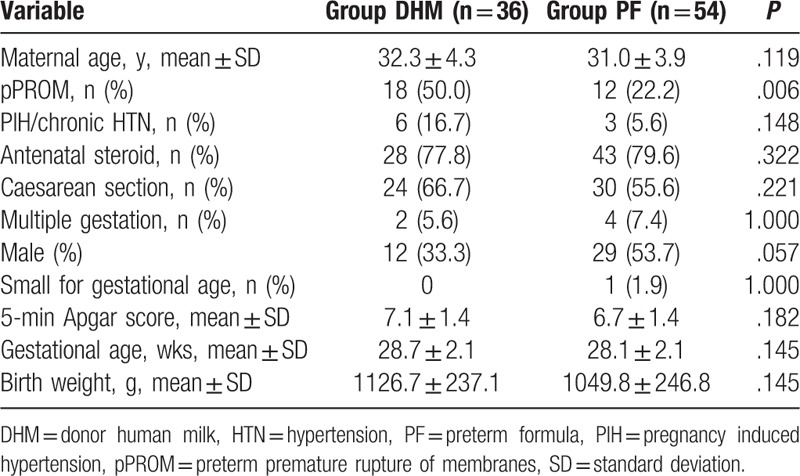
Perinatal characteristics in the donor human milk and preterm formula groups.

The early-onset morbidity and clinical characteristics in the DHM and PF groups are compared in Table 3. There were no significant differences in the use of surfactant, administration of initial antibiotics, and early-stage morbidity after birth such as pneumothorax and treatment of patent ductus arteriosus. There was no additional antibiotic treatment in the DHM group without infection, except for prophylactic antibiotics immediately after birth. Compared with the DHM group, preterm infants in the PF group were more likely to have a long duration of ventilator care, central venous catheter, and a late postnatal age of achieving feeding of 50 and 130 mL/kg/d, longer hospitalization periods, and higher PMA at discharge.

**Table 3:**
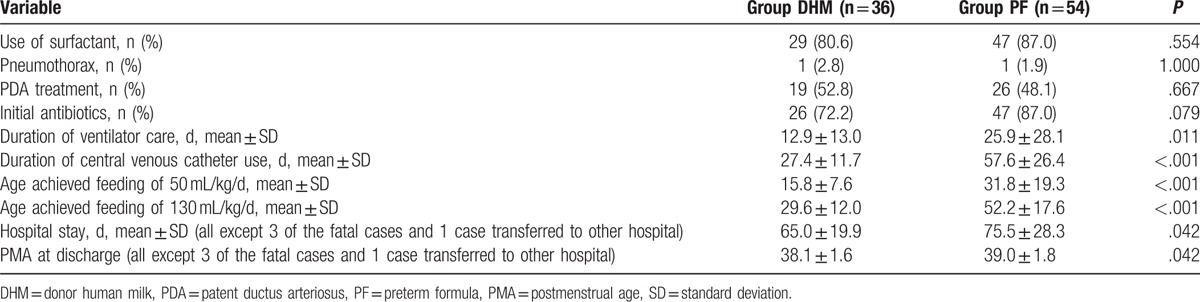
Clinical variables in the donor human milk and preterm formula groups.

### 3.2 Infection-related events and late-onset morbidities in the DHM and PF groups during hospitalization

Table 4 shows the unadjusted and adjusted ORs for LOS, NEC (≥stage 2), BPD, ROP (≥stage 2), severe IVH (≥grade III), and death. The incidence of NEC (≥stage 2) or death was lower in the DHM group, but the incidence was 0 and the OR could not be estimated. The incidence of LOS, LOS and/or NEC (≥stage 2), BPD, and moderate to severe BPD was significantly lower in the DHM in both unadjusted and adjusted analyses; the latter was performed by entering potential confounding variables (sex, GA, pPROM, and 5-minute Apgar score) into the multivariate logistic regression model. There were no differences between the 2 groups with respect to ROP (≥stage 2) and severe IVH. *Staphylococcus epidermidis* infection was the most common cause of LOS (44.4%), followed by infection with *Klebsiella* species (27.8%), *Staphylococcus capitis* (11.1%), and a case each of infection with *Staphylococcus hominis*, *Staphylococcus aureus*, *Escherichia coli*, and *Candida* species (Table 5).

**Table 4:**
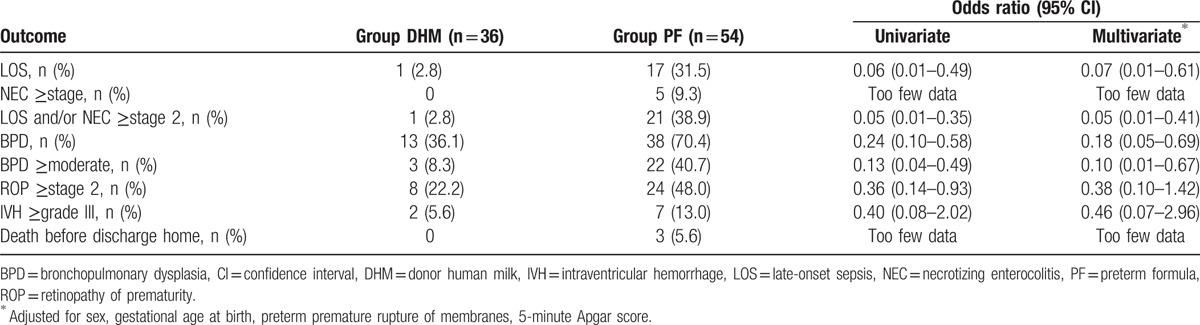
Unadjusted and adjusted odds ratios for the study subjects.

**Table 5:**
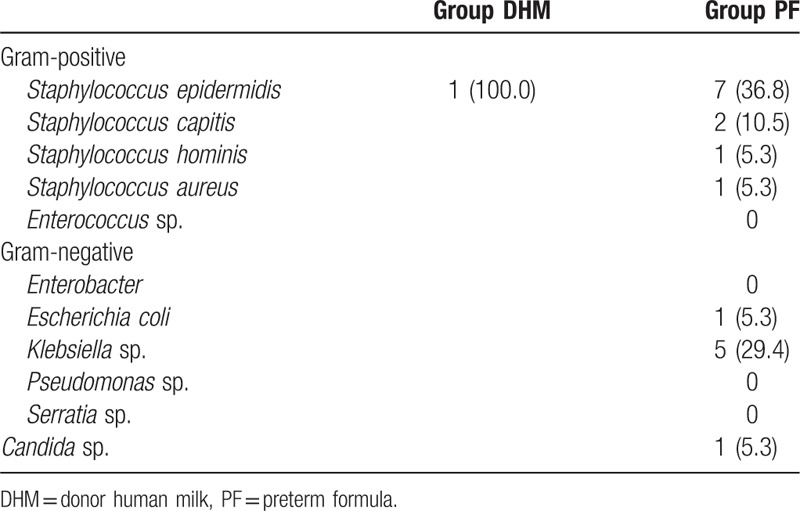
Bacterial and fungal isolates from blood.

### 3.3 The change of growth parameters from birth to 36 weeks’ PMA

**Figure 2:**
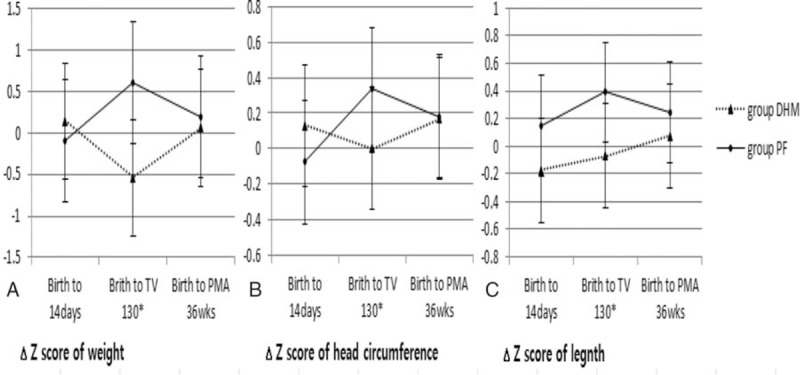
The z-score change of growth parameters for birth to postnatal age 14 days, age achieved feeding of 130 mL/kg/d and postmenstrual age (PMA) 36 weeks: (A) weight, (B) head circumference, (C) height. Data are presented as mean ± SD (^∗^*P* < .05).

The growth parameters in the DHM and PF groups are compared in Table 6. At birth, there were no differences in BW, HC, and height. Compared with infants in the PF group, infants in the DHM group were more likely to have a lower weight (1443.6 ± 381.5 vs 1985.2 ± 373.5 g), HC (27.9 ± 2.4 vs 29.1 ± 1.9 cm), and height (39.7 ± 2.3 vs 42.4 ± 2.5 cm), at the age achieved, after feeding of 130 mL/kg/d. After the commencement of nutrition support, however, growth increased, with the values of the weight, HC, and height at 36 weeks’ PMA, being similar in both groups. The z-score demonstrated that the change in all growth parameters of the infants from birth to the age at which full enteral nutrition of 130 mL/kg/d was achieved was significantly different between both groups. However, there was no significant difference in the growth of the infants from birth to 36 weeks’ PMA, in both groups (Fig. 2).

**Table 6:**
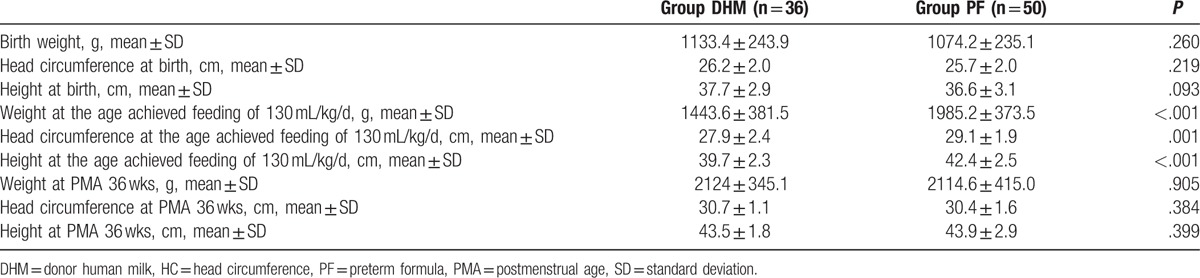
Comparison of growth parameters in the donor human milk and preterm formula groups (all except 3 of the fatal cases and 1 case transferred to other hospital).

## 4 Discussion

The objective of this study was to compare the morbidities and the growth of premature infants who were fed exclusively with DHM, to premature infants who were fed a combination of PF and DHM or HM from birth, until full enteral nutrition was achieved (130 mL/kg/d) in both groups. While the optimal timing of fortification during feeding advancement has not been established, generally fortification takes place between half to full enteral feeds (80 and 160 mL/kg/d) in very-low BW (VLBW) infants.^[19–21]^ It took about 4 weeks to reach 130 ml/kg/d in the DHM group in our NICU. Because NEC occurs more frequently within the first 2 to 4 weeks of life,^[22]^ fortification was initiated after enteral feeding was achieved at 130 ml/kg/d to reduce the risk of NEC.

No significant differences were found in perinatal characteristics and early-onset morbidity, except for the incidence of pPROM between the 2 groups. In the presence of pPROM, the higher incidence of neonatal early-onset sepsis and pneumonia from intrauterine infection has contributed to an increase in neonatal mortality rate.^[23]^ However, the presence of an infection was directly associated with chorioamnionitis.^[24]^

Although numerous studies have demonstrated that feeding PF produces a more favorable result than HM or DM or fortified HM in terms of growth, in VLBW infants, there are still concerns about the increased risks of NEC, increased duration of PN, increased rates of LOS, and BPD in infants receiving PF.^[6,25,26]^ Since NEC occurs more frequently in preterm infants on enteral nutrition, within the first 2 to 4 weeks of life, and occasionally in neonates who have never been fed, the type of milk administered in this stage is very important if NEC must be prevented. The timing of feeds, their volume, and the rate of progression of the volume of feeds should also be considered.^[27–30]^ Our NICU adheres to strict quality assurance guidelines for microbial testing of breast milk, to ensure that the resulting DHM or PM administered to VLBW infants are free of cytomegalovirus (CMV). The breast milk of mothers of preterm infants is also tested using a CMV polymerase chain reaction, to prevent potential CMV infection by their mother's breast milk. Relatively, more infants were fed DHM exclusively for some period after birth, in our NICU. In a randomized controlled trial conducted by Schanler et al,^[31]^ infants fed fortified DHM had similar rates of LOS and NEC, and other infection-related events with infants fed PF. On this basis, to avoid the risk of NEC, we continued to feed the infants with unfortified DHM or HM, until full enteral nutrition was achieved. Several studies have demonstrated that preterm infants fed with DHM have a reduced risk, incidence, and severity of NEC, and a shorter duration of PN than in those fed with PF. Exclusive breastfeeding decreases preterm mortality and incidence of LOS and BPD.^[28,32–34]^ In this study, preterm infants in the DHM group were more likely to have a short duration of a postnatal age achieved feeding of 130 mL/kg/d and ventilator care, which allowed them to have short duration of central venous catheter insertion and reduced lung injury compared with the PF group. Central venous catheter and invasive ventilation are known to affect the development of LOS and BPD in preterm infants.^[35,36]^ In our study, there was significant difference in the incidence of LOS and BPD between both groups. It has been observed that the incidence of infection in the DHM group is reduced, but this should be considered as a possible bias due to the small sample size. Although NEC was lower in the DHM group (0% vs 9.3%), we could not estimate the OR of NEC between the 2 groups because NEC did not occur in the DHM group. Further research with larger samples are required to support and strengthen the accuracy, or otherwise, of this finding.

The preterm infants in the DHM group were more likely to achieve full feeding (130 mL/kg/d) within a shorter period (29.6 ± 12.0 vs 52.2 ± 17.6 days). Few studies have demonstrated that early transition from PN to full enteral feeding might impair in-hospital growth; poor weight gain in the early postnatal period could be associated with the development of in-hospital morbidities, such as LOS, ROP, IVH, BPD, and NEC.^[6,37–39]^ Preterm infants in the DHM group who obviously transitioned early to full enteral feeding, demonstrated restricted growth (weight, height) in the early postnatal period. However, morbidity in this group decreased significantly. Poor weight gain, although not a disease, may result in numerous medical conditions in preterm infants. DHM is obtained usually from women who deliver at term, and donate their milk several months after delivery. As a result, DHM has been proven to contain a low protein and fat content, and many bioactive molecules compared to HM or PF.^[40]^ In our study, the cause of poor weight gain in the early postnatal period may have been because of the low caloric content of the DHM. DHM may have contributed to the reduction in morbidities by enhancing early enteral nutrition and reducing possible inflammatory complications, associated with prolonged total PN.^[41,42]^ As standard protocol performed in this study, these differences catch up to 36 weeks’ PMA. After initial high-calorie nutritional support, the growth rate is increased in the DHM group, and the difference between body weight and HC at 36weeks’ PMA is not significant in both groups.

This study is limited by its small sample size, lack of evaluation of long-term outcomes of neurodevelopmental or cognitive functions after NICU discharge, and the lack of comparison with preterm infants exclusively breastfed with their own mother's milk. Premature infants need vigorous nutritional support for optimal growth in the neonatal period than at any other time of their life.^[43]^ There is a definite need for long-term follow-up of neurodevelopmental function after discharge.

## 5 Conclusions

In conclusion, exclusive DHM feeding in the early postnatal period can provide protective benefits from several morbidities such as LOS/NEC and BPD, and encourage successful catch-up growth at 36 weeks’ PMA, after high-calorie nutritional support in VLBW infants. Therefore, in situations where mother's own breast milk is unavailable, DHM can be the first choice of an alternative source of nutrition in preterm infants.
